# Author Correction: Using rare genetic mutations to revisit structural brain asymmetry

**DOI:** 10.1038/s41467-024-47545-5

**Published:** 2024-04-10

**Authors:** Jakub Kopal, Kuldeep Kumar, Kimia Shafighi, Karin Saltoun, Claudia Modenato, Clara A. Moreau, Guillaume Huguet, Martineau Jean-Louis, Charles-Olivier Martin, Zohra Saci, Nadine Younis, Elise Douard, Khadije Jizi, Alexis Beauchamp-Chatel, Leila Kushan, Ana I. Silva, Marianne B. M. van den Bree, David E. J. Linden, Michael J. Owen, Jeremy Hall, Sarah Lippé, Bogdan Draganski, Ida E. Sønderby, Ole A. Andreassen, David C. Glahn, Paul M. Thompson, Carrie E. Bearden, Robert Zatorre, Sébastien Jacquemont, Danilo Bzdok

**Affiliations:** 1 Mila - Québec Artificial Intelligence Institute, Montréal, QC Canada; 2 Department of Biomedical Engineering, Faculty of Medicine, McGill University, Montréal, QC Canada; 3 Centre de recherche CHU Sainte-Justine, Montréal, QC Canada; 4 LREN - Department of Clinical Neurosciences, Centre Hospitalier Universitaire Vaudois and University of Lausanne, Lausanne, Switzerland; 5 Imaging Genetics Center, Stevens Neuroimaging and Informatics Institute, Keck School of Medicine of USC, Marina del Rey, CA USA; 6 Institut universitaire en santé mentale de Montréal, University of Montréal, Montréal, QC Canada; 7 Department of Psychiatry, University of Montreal, Montréal, QC Canada; 8 Semel Institute for Neuroscience and Human Behavior, Departments of Psychiatry and Biobehavioral Sciences and Psychology, UCLA, Los Angeles, CA USA; 9 School for Mental Health and Neuroscience, Maastricht University, Maastricht, Netherlands; 10 Centre for Neuropsychiatric Genetics and Genomics, Cardiff University, Cardiff, UK; 11 Division of Psychological Medicine and Clinical Neurosciences, School of Medicine, Cardiff University, Cardiff, UK; 12 Neuroscience and Mental Health Innovation Institute, Cardiff University, Cardiff, UK; 13 Neurology Department, Max-Planck-Institute for Human Cognitive and Brain Sciences, Leipzig, Germany; 14 NORMENT, Division of Mental Health and Addiction, Oslo University Hospital and University of Oslo, Oslo, Norway; 15 Department of Medical Genetics, Oslo University Hospital, Oslo, Norway; 16 KG Jebsen Centre for Neurodevelopmental Disorders, University of Oslo, Oslo, Norway; 17 Department of Psychiatry, Boston Children’s Hospital and Harvard Medical School, Boston, MA USA; 18 International Laboratory for Brain, Music and Sound Research, Montréal, QC Canada; 19 Department of Pediatrics, University of Montréal, Montréal, QC Canada; 20 TheNeuro - Montreal Neurological Institute (MNI), McConnell Brain Imaging Centre, Faculty of Medicine, McGill University, Montréal, QC Canada

**Keywords:** Genetics of the nervous system, Genome-wide association studies, Neurodevelopmental disorders

Correction to: *Nature Communications* 10.1038/s41467-024-46784-w, published online 26 March 2024

In this article the affiliation details for Robert Zatorre were incorrectly given as ‘Department of Pediatrics, University of Montréal, Montréal, Quebec, Canada.’ but should have been ‘TheNeuro - Montreal Neurological Institute (MNI), McConnell Brain Imaging Centre, Faculty of Medicine, McGill University, Montreal, QC, Canada’. The original article has been corrected.

