# Supplementary material for: Using rare genetic mutations to revisit structural brain asymmetry
Source: Nat Commun. 2024 Mar 26;15:2639. doi: 10.1038/s41467-024-46784-w (PMC10966068; doi:10.1038/s41467-024-46784-w)
Supplement: Supplementary file 1 — Supplementary Information [file 41467_2024_46784_MOESM1_ESM.pdf]

# Supplementary Figures

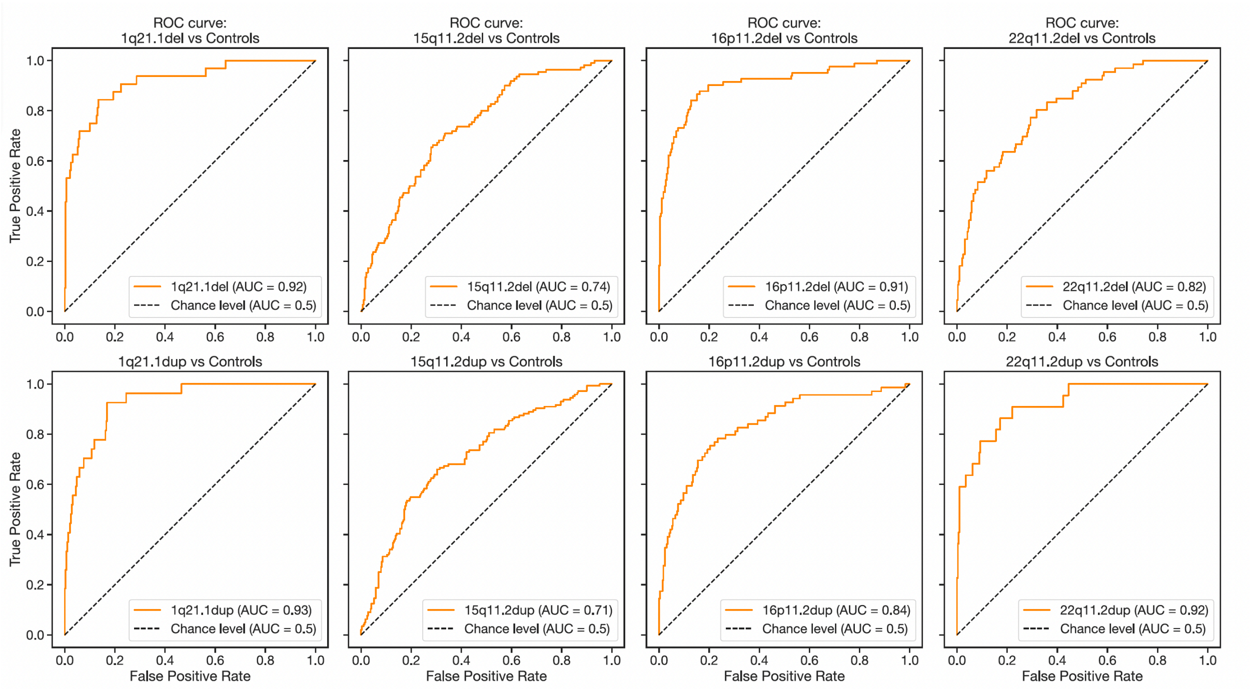


Supplementary Figure 1

**All eight CNVs can well separate CNV from control participants based on brain-imaging features.**

We leverage the ROC curve (receiver operating characteristic curve) analysis in order to evaluate the discrimination performance of eight LDA models. Each LDA model was aimed at classifying between dedicated CNVs and controls. The depicted ROC curve is a plot of the True Positive Rate (sensitivity) on the y-axis against the False Positive Rate (1−specificity) on the x-axis for varying values of the threshold. The AUC (Area under the ROC Curve) metric can serve as a single-number summary of model fit. Across the eight analyzed CNVs, the maximal AUC was 0.93 (1q21.1dup), while the minimal AUC was 0.71 (15q11.2dup). All AUC values are well above the chance level (AUC=0.5), suggesting a good model fit for all eight LDA models.

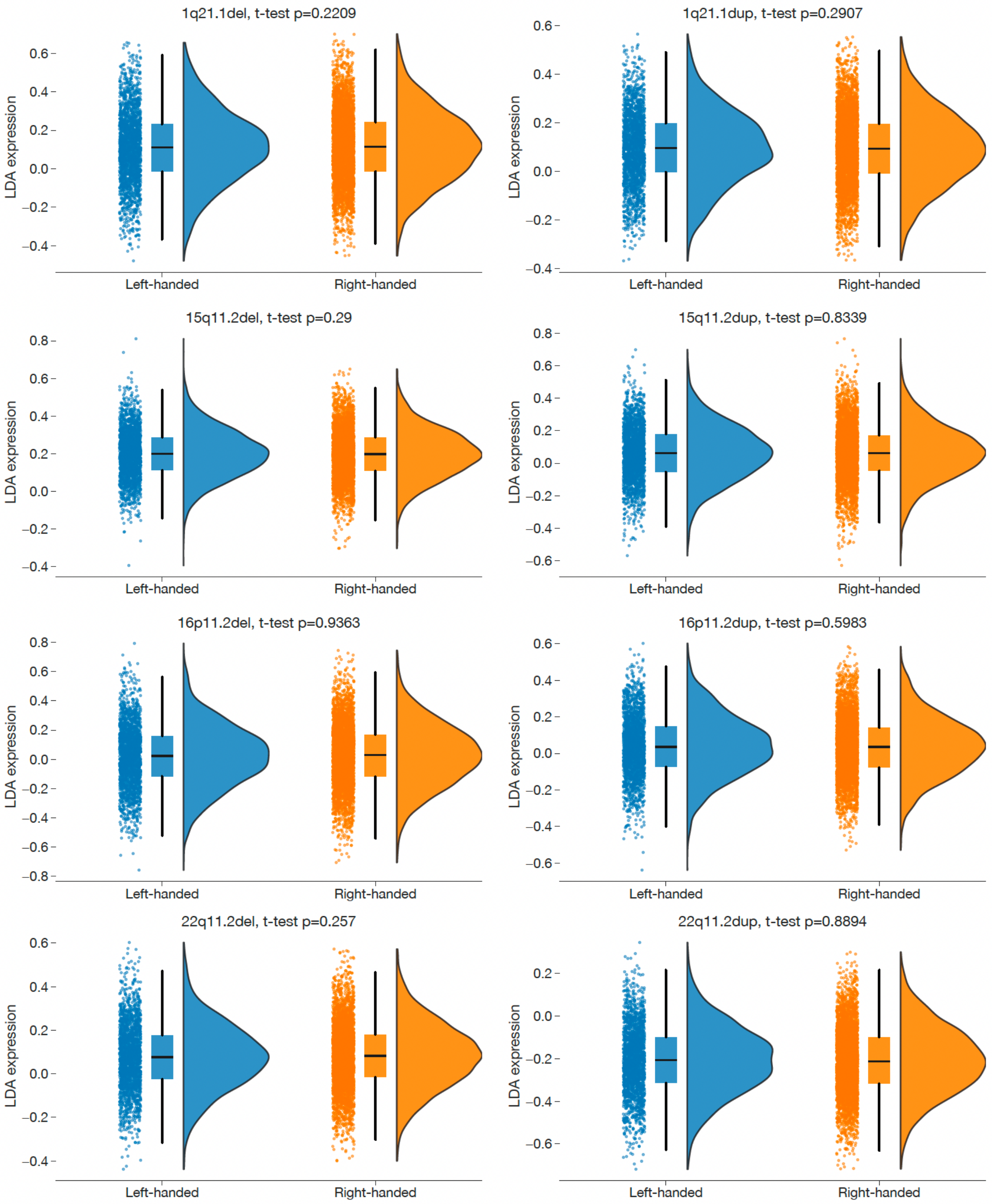


Supplementary Figure 2

**CNV-specific LDA models are not associated with handedness in the UK Biobank sample**

We performed an analysis directed at the association of derived brain asymmetry patterns and handedness in the UK Biobank sample. Specifically, for each of the eight LDA models dedicated to eight CNVs, we quantified LDA expression for each of the 36,000 UK Biobank participants used in this study. This expression is computed as a weighted sum of regional LDA coefficients and participant's regional asymmetries. In the next step, we used a t-test to compare if the LDA expressions differ between left- and right-handed participants. We did not find a significant difference in LDA expression depending on handedness for any of the eight CNVs.

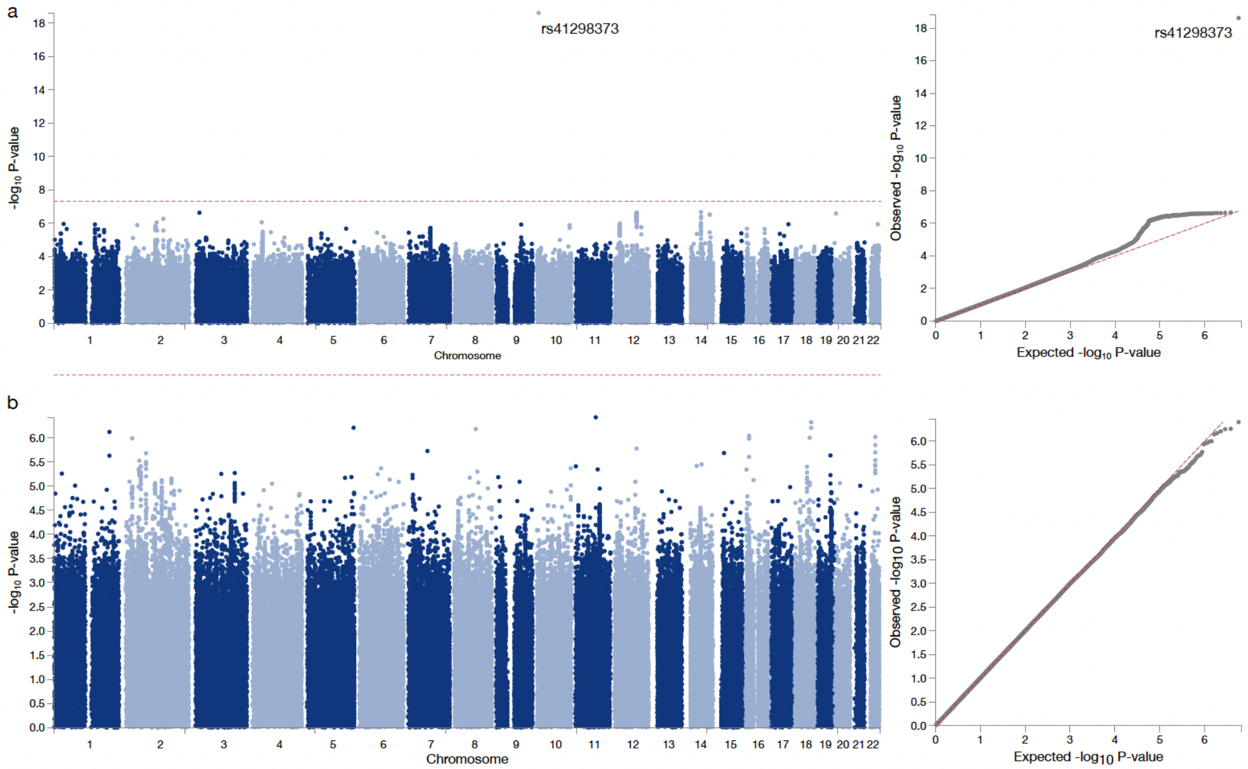


Supplementary Figure 3

**The influence of directionality of planum temporale asymmetry on GWAS results**

Our GWAS of planum temporale asymmetry revealed a single significantly associated SNP: rs41298373. Since planum temporale asymmetry is a directed measure, we investigated whether this directionality influences GWAS results. Specifically, we conducted two additional GWAS in the same pool of 30,358 UK Biobank subjects directed at a) the absolute value of planum temporale asymmetry and b) the absolute value of across-subjects z-scored planum temporale asymmetry. While computing an absolute value did not reveal additional SNPs, the use of an absolute z-score asymmetry index led to zero significantly associated SNPs. Therefore, different definitions of the asymmetry index did not highlight new genomic loci.

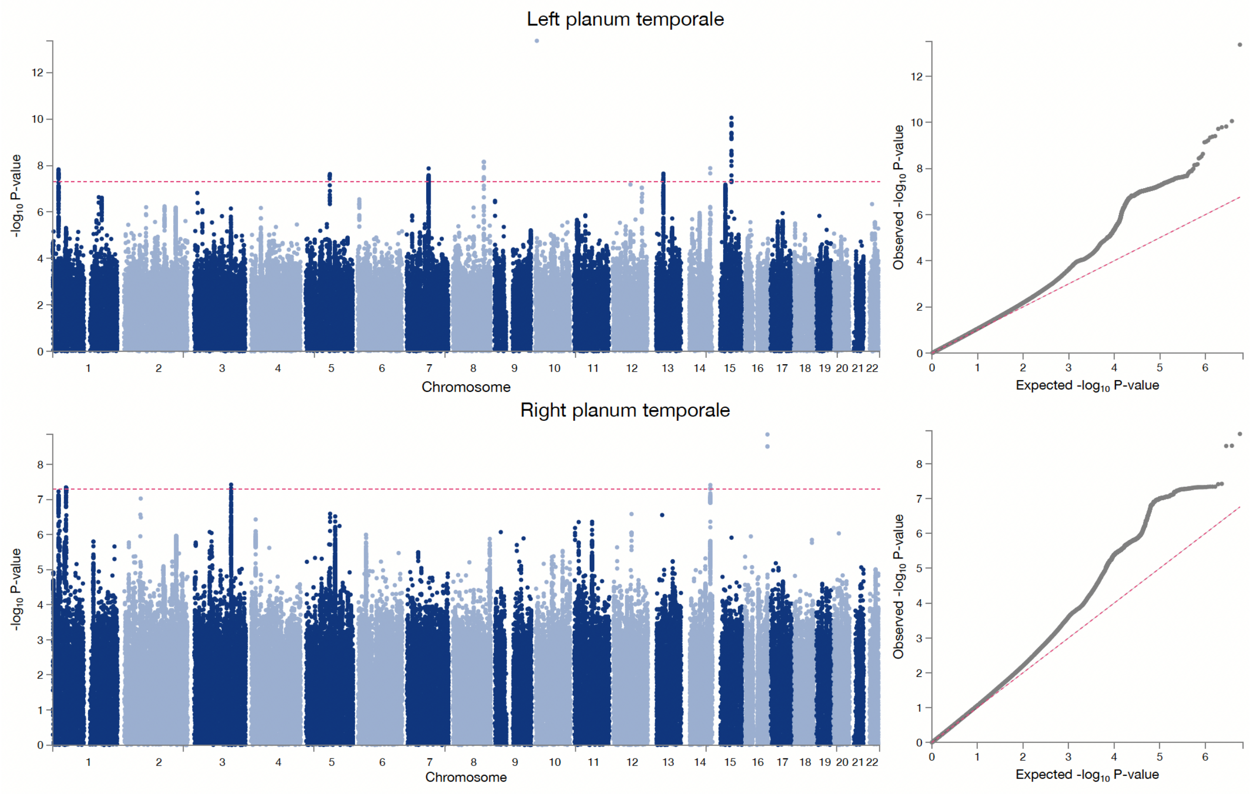


Supplementary Figure 4

**Dissecting the impact of common genetic variants on left and right planum temporale**

We conducted a GWAS (genome-wide association study) separately for the left and right planum temporale volume asymmetry in the 30,358 UK Biobank subjects. Based on GWAS of the left planum temporale volume, we found 726 significant candidate SNPs that mapped to eight genomic loci. Our GWAS of the right planum temporale volume yielded 368 candidate SNPs in four genomic loci. The QQ plots associated with both analyses suggest that genetic studies with more participants will likely locate additional loci.

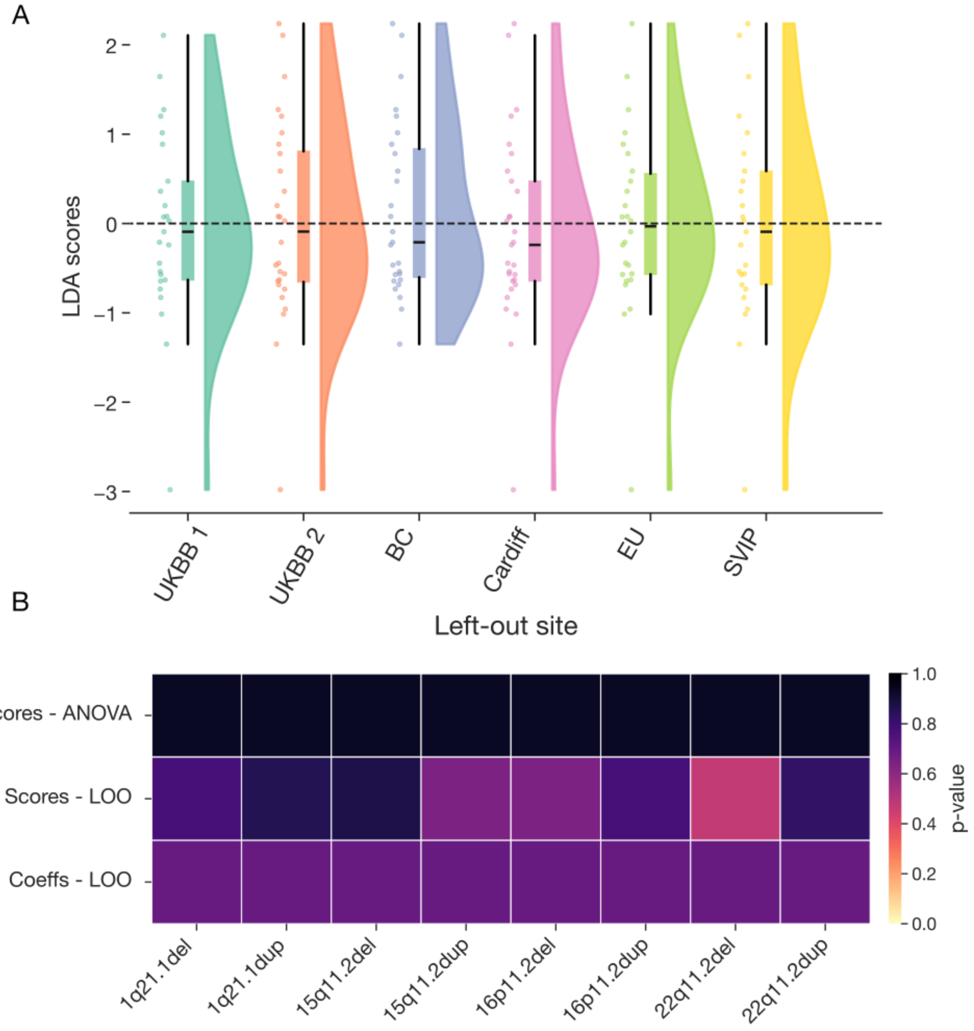


Supplementary Figure 5

**Low-dimensional summaries of brain-imaging features do not depend on the recruitment site for any of the eight CNVs**

We conducted two sensitivity analyses dedicated to probe the effect of the recruitment site on the derived LDA scores separately for each CNV. A) Leave-one-site-out analysis. For a single CNV (here 1q21.2 deletion), we removed the LDA scores of subjects recruited at a given site (i.e., Cardiff). This reduced set of LDA scores is depicted using the raincloud plot, which combines a scatter plot, a box plot (whiskers equal to 1.5 times the interquartile range), and a violin plot. We then compared the remaining LDA scores with the original LDA scores of all subjects carrying the CNV using a two-sample t-test. Such procedure was repeated for every site, and minimal p-value across sites was used to quantify the effects of the recruitment site on the LDA scores for a given CNV. B) Results of sensitivity analyses. The second sensitivity analysis consisted of employing one-way ANOVA separately for each CNV. Finally, we also repeated leave-one-site-out analysis to examine the stability of LDA coefficients. For each CNV, we removed the asymmetry measurements of subjects recruited at a given site. This reduced set of participants was used to derive LDA coefficients. We then compared the new set of LDA coefficients with the original LDA coefficients of all subjects carrying the CNV using a two-sample t-test. Such procedure was repeated for every site, and minimal p-value across sites was used to quantify the effects of the recruitment site on the LDA coefficients for a given CNV. All resulting p-values were corrected from multiple comparisons using

the FDR procedure across all CNVs separately for each analysis. As displayed in the heatmap, we did not observe a significant relationship between LDA scores or coefficients and a site for any of the CNVs. Collectively, our set of sensitivity analyses demonstrated that the recruitment site did not drive the obtained LDA solutions.

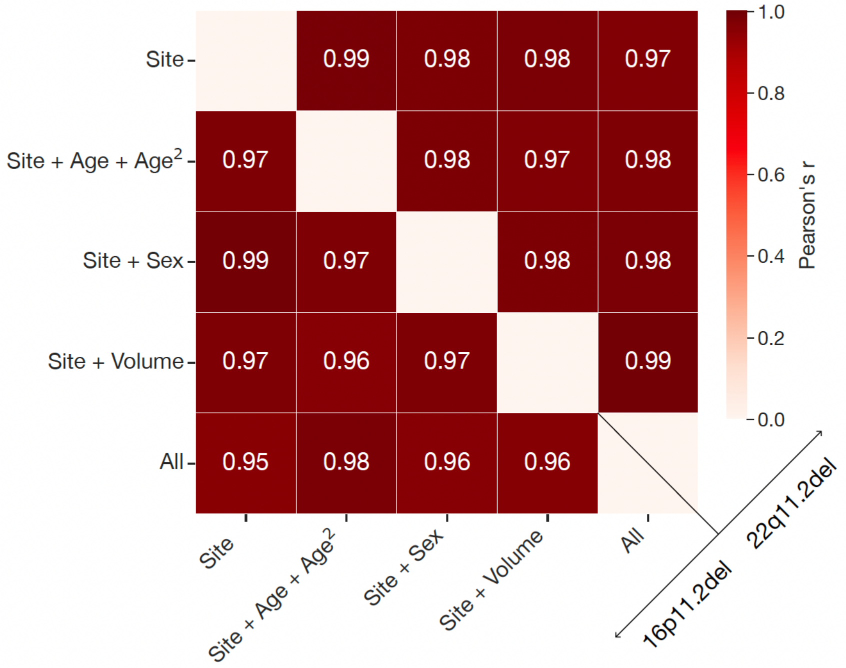


Supplementary Figure 6

**The effects of deconfounding factors on extracted patterns of brain asymmetry**

In our study, all derived regional brain volumes were adjusted for variation that can be explained by the scanning site. To probe the effect of other confounding variables, we further adjusted regional brain volumes for the effects of intracranial volume, age and age$^2$, sex, and all of these variables together. In the next step, we compared LDA asymmetry patterns derived using asymmetry indices based on these different preprocessing scenarios. We probed the influence of preprocessing on LDA models associated with both 16p11.2 and 22q11.2 deletion. Since all Pearson's correlations were > 0.95, adding more deconfounding variables did not influence the resulting asymmetry patterns for any CNV.

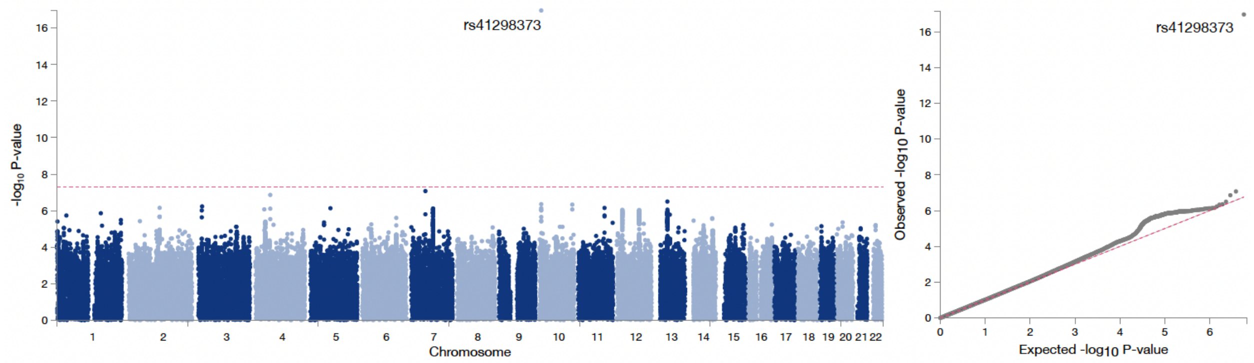


Supplementary Figure 7

**Genetic analysis of planum temporale with additional covariates**

To complement our original GWAS of planum temporale, we studied the influence of including total brain volume as an additional covariate. This preprocessing step did not lead to any change as the rs41298373 remained the only significantly associated SNP.
