# Supplementary material for: Using rare genetic mutations to revisit structural brain asymmetry
Source: Nat Commun. 2024 Mar 26;15:2639. doi: 10.1038/s41467-024-46784-w (PMC10966068; doi:10.1038/s41467-024-46784-w)
Supplement: Supplementary file 3 — Reporting Summary [file 41467_2024_46784_MOESM3_ESM.pdf]

nature portfolio

Corresponding author(s): Danilo Bzdok

Last updated by author(s): Jan 9, 2024

Reporting Summary

Nature Portfolio wishes to improve the reproducibility of the work that we publish. This form provides structure for consistency and transparency in reporting. For further information on Nature Portfolio policies, see our Editorial Policies and the Editorial Policy Checklist.

Statistics

For all statistical analyses, confirm that the following items are present in the figure legend, table legend, main text, or Methods section.

n/a	Confirmed	
☐	☒	The exact sample size (*n*) for each experimental group/condition, given as a discrete number and unit of measurement
☐	☒	A statement on whether measurements were taken from distinct samples or whether the same sample was measured repeatedly
☐	☒	The statistical test(s) used AND whether they are one- or two-sided<br>*Only common tests should be described solely by name; describe more complex techniques in the Methods section.*
☐	☒	A description of all covariates tested
☐	☒	A description of any assumptions or corrections, such as tests of normality and adjustment for multiple comparisons
☐	☒	A full description of the statistical parameters including central tendency (e.g. means) or other basic estimates (e.g. regression coefficient) AND variation (e.g. standard deviation) or associated estimates of uncertainty (e.g. confidence intervals)
☐	☒	For null hypothesis testing, the test statistic (e.g. *F*, *t*, *r*) with confidence intervals, effect sizes, degrees of freedom and *P* value noted<br>*Give P values as exact values whenever suitable.*
☐	☒	For Bayesian analysis, information on the choice of priors and Markov chain Monte Carlo settings
☐	☒	For hierarchical and complex designs, identification of the appropriate level for tests and full reporting of outcomes
☐	☒	Estimates of effect sizes (e.g. Cohen's *d*, Pearson's *r*), indicating how they were calculated

Our web collection on statistics for biologists contains articles on many of the points above.

Software and code

Policy information about availability of computer code

Data collection	No data were collected for this study as all analyses were conducted on data that were already collected as part of several large scale consortium data sets.
Data analysis	The processing scripts and custom analysis software used in this work are available in a publicly accessible GitHub repository with instructions on how to set up a similar computation environment and with examples of key visualizations in the paper:<br>https://github.com/surchs/Neuropsychiatric_CNV_code_supplement<br>https://github.com/dblabs-mcgill-mila/CNV-asymetry<br>Software used for data analysis:<br>scikit-learn (version: 1.3.2)<br>numpy (version: 1.21.5)<br>seaborn (version: 0.13.0)<br>statsmodels (version: 0.14.0)<br>scipy (version: 1.11.3)<br>nibabel (version: 5.1.0)<br>enigmatoolbox(version: 2.0.3)<br>joypy (version: 0.2.4)<br>pysankey (version: 0.1.0)<br>pandas (version: 1.3.5)<br>matplotlib (version: 3.5.1)<br>SUITPy (version: 1.3.2)

nilearn (version: 0.10.2)
MATLAB (version: R2018b)
SPM12 (version: r7771)
FMRIB automatic segmentation tool (part of FMRIB Software Library v6.0)
FNIRT (part of FMRIB Software Library v6.0)
FAST (part of FMRIB Software Library v6.0)
MCFLIRT (part of FMRIB Software Library v6.0)
Brain Extraction Tool (part of FMRIB Software Library v6.0)
Python (version: v3.8.12)
neurosynth (version: v0.3.8)

For manuscripts utilizing custom algorithms or software that are central to the research but not yet described in published literature, software must be made available to editors and reviewers. We strongly encourage code deposition in a community repository (e.g. GitHub). See the Nature Portfolio guidelines for submitting code & software for further information.

Data

Policy information about availability of data

All manuscripts must include a data availability statement. This statement should provide the following information, where applicable:

- Accession codes, unique identifiers, or web links for publicly available datasets
- A description of any restrictions on data availability
- For clinical datasets or third party data, please ensure that the statement adheres to our policy

The majority of 16p11.2 data are publicly available (https://www.sfari.org/). For the 22q11.2 sample, raw data are available upon request from the PI (CB). All derived measures used in this study are available upon request (SJ). The rest of the CNV carriers' data cannot be shared as participants did not provide consent. All data from UK Biobank are available to other investigators online (ukbiobank.ac.uk). The Harvard-Oxford and Diedrichnsen atlases are accessible online (http://fsl.fmrib.ox.ac.uk/fsl/fslwiki/Atlases).

Research involving human participants, their data, or biological material

Policy information about studies with human participants or human data. See also policy information about sex, gender (identity/presentation), and sexual orientation and race, ethnicity and racism.

Reporting on sex and gender	We used the largest multi-site clinical dataset of participants carrying rare CNVs. These participants were recruited only based on the presence of CNVs. Moreover, we identified the full set of ~40,000 UK Biobank participants with structural MRI images without exclusions. Among these participants, 48% were men and were 52% women. Our analysis critically depended on large sample size, and therefore we were not able to perform gender and/or sex-based analyses.
Reporting on race, ethnicity, or other socially relevant groupings	See above.
Population characteristics	We pooled data from 5 different cohorts: Cardiff University (UK), 16p11.2 European Consortium (Lausanne, Switzerland), University of Montreal (Canada), UCLA (Los Angeles, USA) and the Variation in individuals Project (SVIP, USA). Detailed information are available in Modenato et al., 2021. Among the UK Biobank participants, 48% were men and were 52% women with age between 40 and 69 y.o. when recruited [mean age 55 y.o., standard deviation (SD) 7.5 y.]). Models were all adjusted for sex, site, head motion, total intracranial volume and age. Information about all subjects are available in Table 1.
Recruitment	Clinically ascertained CNV carriers were recruited as either probands referred for genetic testing, or as relatives. UK Biobank recruitment was done as part of the UKbiobank initiative by flyers and other means common in epidemiological research. For details on representativeness see Frey et al., 2017.
Ethics oversight	Signed consents were obtained from all clinical participants or legal representatives prior to the investigation. The current study, which is purely analytical was approved by the IRB (Project 4165) of the Sainte Justine Hospital. UK Biobank participants gave written, informed consent for the study, which was approved by the Research Ethics Committee. The present analyses were conducted under UK Biobank application number 25163. Further information on the consent procedure can be found here (biobank.ctsu.ox.ac.uk/crystal/field.cgi?id=200).

Note that full information on the approval of the study protocol must also be provided in the manuscript.

Field-specific reporting

Please select the one below that is the best fit for your research. If you are not sure, read the appropriate sections before making your selection.

[x] Life sciences [ ] Behavioural & social sciences [ ] Ecological, evolutionary & environmental sciences

For a reference copy of the document with all sections, see nature.com/documents/nr-reporting-summary-flat.pdf

Life sciences study design

All studies must disclose on these points even when the disclosure is negative.

Sample size	Our clinical dataset is one of the largest CNV datasets today with brain scans from 842 subjects: 552 CNV carriers and 290 controls not carrying any CNV. Previously published studies have shown that 16p11.2 and 22q11.2 deletions have large effect-sizes >0.8 to 1 Cohen's d that have been detected with samples of 20 individuals and more. Further, we used all subject data from the 40,000 UK Biobank release, no exclusions. Previous studies using the same sample size documented detectable and robust effect sizes for the selected CNVs (e.g. Kopal et al, 2023).
Data exclusions	We used MRI data from all the participants previously published in Modenato et al., 2021 where preprocessed data were visually controlled for quality of the co-registration, head motion, and related artifacts by one rater. We identified the full set of ~40,000 UK Biobank participants who had structural MRI image and passed quality control, without exclusions.
Replication	All models were evaluate using in-sample ROC curves and, specifically, area under the ROC curve.<br>All bootstrap test were performed with 10,000 re-samplings.<br>All replication tests were succesfull and proved the robusstness of chosen solution.
Randomization	Randomization was not relevant to our study as there were no experimental manipulations. Participants did not receive any treatment.
Blinding	Blinding was not relevant to our study. Blinding is withholding information about treatment assignment. However, there was no treatment or experimental manipulation in our study.

Reporting for specific materials, systems and methods

We require information from authors about some types of materials, experimental systems and methods used in many studies. Here, indicate whether each material, system or method listed is relevant to your study. If you are not sure if a list item applies to your research, read the appropriate section before selecting a response.

Materials & experimental systems

n/a	Involved in the study	
☒	☐	Antibodies
☒	☐	Eukaryotic cell lines
☒	☐	Palaeontology and archaeology
☒	☐	Animals and other organisms
☒	☐	Clinical data
☒	☐	Dual use research of concern
☒	☐	Plants

Methods

n/a	Involved in the study	
☒	☐	ChIP-seq
☒	☐	Flow cytometry
☐	☒	MRI-based neuroimaging

Plants

Seed stocks	N/A
Novel plant genotypes	N/A
Authentication	N/A

Magnetic resonance imaging

Experimental design

Design type	Please see "Methods" for full details. Our analyses include data from Structural MRI (T1).
Design specifications	MRI data processing (to generate imaging-derived phenotypes) was done previously and is full described in references (Miller et al. 2016 and Modenato et al. 2021).
Behavioral performance measures	Behavioral performance in the MRI scanner was not used in this study.

Acquisition

Imaging type(s)	Please see "Methods" for full details. Our analyses include data from Structural MRI (T1).
Field strength	3 T
Sequence & imaging parameters	MRI data acquisition for the structural covers several pages of full detail, which is fully provided previously in Miller et al. 2016 and Modenato et al. 2021.
Area of acquisition	Siemens' auto-align was used to include the full brain in the imaged field-of-view; this was checked (and corrected if necessary) by the radiographer.

Diffusion MRI ☐ Used ☒ Not used

Preprocessing

Preprocessing software	See above (covered previously in full detail in Miller et al. 2016 and Modenato et al. 2021).
Normalization	See above (covered previously in full detail in Miller et al. 2016 and Modenato et al. 2021).
Normalization template	MNI 152
Noise and artifact removal	See above (covered previously in full detail in Miller et al. 2016 and Modenato et al. 2021).
Volume censoring	No censoring was performed.

Statistical modeling & inference

Model type and settings	N/A
Effect(s) tested	N/A

Specify type of analysis: ☐ Whole brain ☒ ROI-based ☐ Both

Anatomical location(s): The Harvard-Oxford and Diedrichnsen atlases with 96 cortical, 14 subcortical, and 20 cerebellar volume measures.

Statistic type for inference (See Eklund et al. 2016)	Our analysis was conducted using linear discriminant analysis of all 65 brain regional asymmetry indices.
Correction	FDR corrections were used for testing linear association strength when applicable.

Models & analysis

n/a	Involved in the study
☒	☐ Functional and/or effective connectivity
☒	☐ Graph analysis
☐	☒ Multivariate modeling or predictive analysis

Multivariate modeling and predictive analysis	We used Linear Discriminant Analysis for asymmetry of structural MRI data.
